# Developing a COVID-19 module for the European Social Survey

**DOI:** 10.1186/s42409-021-00029-4

**Published:** 2021-11-23

**Authors:** Tim Hanson, Marc Helbling, Rahsaan Maxwell, Richard Traunmüller, Kostas Gemenis, Levente Littvay

**Affiliations:** 1 European Social Survey ERIC, City, University of London, London, UK; 2 University of Mannheim, Mannheim, Germany; 3 University of North Carolina, Chapel Hill, USA; 4 Max Planck Institute for the Study of Societies, Cologne, Germany; 5 Central European University, Vienna, Austria

**Keywords:** COVID-19, European Social Survey, Cross-national surveys, Questionnaire design

## Abstract

This short report describes the development of a COVID-19 module for Round 10 of the European Social Survey (ESS). The module is formed of 20 items, including 10 agreed with ESS National Coordinators and 10 developed by two academic teams following an external call for content. The majority of ESS participating countries will field the module for Round 10 fieldwork in 2020–2021. This report provides the rationale for the module, presents challenges of developing such a module for ESS, and gives an overview of the question development and testing process. The two external sub-modules—*Government authority and legitimacy in the age of a pandemic* and *COVID-19 conspiracy beliefs and government rule compliance*—are also introduced.

**Supplementary Information:**

The online version contains supplementary material available at 10.1186/s42409-021-00029-4.

## Introduction to the European Social Survey

The European Social Survey (ESS) is an academically driven cross-national survey measuring attitudes and behaviour across Europe (Fitzgerald & Jowell, 2010). Face-to-face fieldwork has been conducted every two years since 2002, with nine rounds of data collection completed to date. In total, 38 countries have participated in at least one round of the survey, with 15 countries participating in all nine rounds. More than thirty countries are expected to participate at Round 10, in 2020–2021 (European Social Survey European Research Infrastructure Consortium, 2020).

The ESS questionnaire includes a core component, which is largely unchanged between rounds, and two rotating modules. The rotating modules are selected via a competition among academic teams for each round. The average questionnaire length is approximately one hour.

Round 10 fieldwork for ESS was originally scheduled to run from September 2020 to January 2021. However, the COVID-19 pandemic made face-to-face fieldwork for most of this period impossible and it was decided to extend the Round 10 deadline to December 2021. Most countries expect to conduct face-to-face fieldwork in 2021. However, some will be unable to deliver face-to-face fieldwork at any time in 2021 and so will switch to an alternative self-completion method that has been developed as an emergency measure for Round 10 (Hanson, 2021).

## The rationale for including a COVID-19 module

The source questionnaire for Round 10 of ESS was finalised in April 2020, at roughly the time the pandemic was taking hold across Europe. Following consultation with National Coordinating Teams and the ESS Core Scientific Team, it was decided to develop a COVID-19 module. This was offered to participating countries on an opt-in basis as an alternative to the country-specific items normally permitted.

While other studies had compared the impact of the pandemic cross-nationally, the inclusion of a COVID-19 module on ESS offered some important benefits. The ESS employs the highest standards of data collection (see, for example, Wuyts & Loosveldt, 2019), with all countries required to deliver random probability samples to a detailed specification. This meant that users could be confident in the quality of the study and the reliability of the results.

Including a COVID-19 module on ESS would allow data users to analyse these measures alongside hundreds of other items in the ESS questionnaire. For Round 10, this included two rotating modules that were felt to be highly relevant in the context of the pandemic: *Understanding and Evaluations of Democracy* and *Digital Social Contacts in Work and Family Life*.

It was also felt that the inclusion of measures relating to COVID-19 would be expected by ESS data users given the rarity and significance of the pandemic and the impact this would have on peoples’ lives across Europe.

The module aimed to reflect the interests of both ESS national teams and external data users. Therefore, it was decided to divide the module (up to 20 items in total) into two parts. The first part (10 items) would be developed with ESS national teams. The second part (10 items) would be drawn from an external call for academic teams. Two mini-modules (5 items each) would be selected from the external call.

## Challenges of developing a COVID-19 module

There were a number of challenges of developing the module in the context of ESS.

First, there was considerable uncertainty over fieldwork timing and variations between countries. One impact of the pandemic was that Round 10 fieldwork may be carried out over a longer period than would usually be expected for an ESS round. This meant that fieldwork dates may differ by up to 12 months between countries, with some aiming to start fieldwork in autumn 2020 and others in autumn 2021. Therefore, any questions that relied on comparing the impact of the pandemic at an identical time between countries would be unsuitable.

Second, the questions needed to be developed during the height of the pandemic, in Spring-Summer 2020. At this stage, there was much focus across a range of studies on the immediate impact of the pandemic.^1^ However, it was expected that by the time ESS fieldwork was possible, the (immediate) impact of the pandemic would have lessened to the extent that life may have returned to some form of normality. While it was therefore clear that the focus needed to be on topics that would have longer-term relevance, it was somewhat unknown what the key debates and questions would be.

Third, an ongoing challenge on ESS is to design questions that are meaningful and can be understood in a comparable way across the range of countries and languages included in the survey. This was also a challenge for the COVID-19 module, especially given differences in impact and policy responses between countries. For example, some topics were seen as a higher priority to cover in some countries than others. Equally, certain responses to the pandemic (e.g. “furlough” schemes) were not always consistent between countries. This required ongoing consultation with the ESS National Coordinators through the development process.

A final challenge was the speed at which the module needed to be developed. ESS’s usual questionnaire development process for rotating modules runs to around 20 months and includes multiple stages of pre-testing.^2^ The COVID-19 module would need to be developed in a period of 2–3 months with limited scope for pre-testing. The resulting data would, however, be published alongside other ESS variables that had undergone much more extensive development, and ESS data users would expect the questions to be developed to ESS’s usual high standards. The challenge, therefore, was to develop high-quality and reliable measures for the COVID-19 module without the time or resources usually available to develop ESS items.

A timetable showing the development period for the module is included in Appendix A.

## Development of items with ESS national teams

As noted above, 10 of the COVID-19 module items would be allocated to and agreed with the participating national teams. This first involved inviting national teams to suggest topics for inclusion, which resulted in a long-list of possible topics. These topics were then narrowed down to priority areas.

Some topics were excluded on the basis of being more focused on short-term aspects of the pandemic (e.g. experience of home teaching; whether people had complied with government rules during lockdowns) or being too complex to properly cover in a short module (e.g. impact of pandemic on mental health; international cooperation in pandemic response). The priority topics were also felt to fit with the core ESS questionnaire content (e.g. satisfaction with and trust in governments) and looked beyond shorter-term impacts of the pandemic. Questions were included on whether people had COVID-19, impacts on employment, and vaccinations, as it was felt data users would see these as important analysis variables to better understand the relationship between experiences of the pandemic and other ESS measures.

The full set of topics proposed by national teams is included in Appendix B. The following topics were considered the highest priority areas which would be developed into questions for the module^3^:

- Whether had COVID-19 (K17, K18)
- Impact of pandemic on employment (K19)
- Satisfaction with overall government response to pandemic (K9)
- Satisfaction with government support for groups particularly affected (K10, K11, K12)
- Satisfaction with health services during pandemic (K13)
- How well government balanced protecting the economy versus protecting people’s health (K14)
- Trust in government to deal with impact of pandemic (K15)
- Willingness to be vaccinated (K20)
- How jobs should be prioritised following the pandemic^4^

Once the topics were selected, draft questions were developed. This involved a review of other surveys^5^ as well as developing new items where necessary. Questions were further developed through an iterative process of review and adaptation between the ESS central design team and national teams.

## Development of external sub-modules

A “call for module proposals” was issued on the ESS website on 22 May 2020, with a deadline of 16 June for submission of proposals. Given the quick turnaround, requirements for proposals were greatly simplified compared with the much more extensive process involved in the development of ESS rotating modules.

Proposals could be a maximum of two pages and needed to cover relevance and originality, methodology, and impact. The proposed set of five questions needed to be provided. The call was open to individual researchers and teams of researchers.

The call document also stated that applicants should consider that ESS data collection would likely take place some time after the initial phase of the pandemic, and that fieldwork dates would vary between countries.

In total, 24 eligible proposals were received and assessed following a two-stage process. The selected sub-modules were:

- Government authority and legitimacy in the age of a pandemic (Helbling et al., 2020).
- COVID-19 conspiracy beliefs and government rule compliance (Gemenis et al., 2020)

The selected sub-modules were both judged to link well with other areas covered by ESS, extending analysis possibilities for data users. For example, the Helbling et al. proposal linked to the rotating Round 10 module on “Understandings and Evaluations of Democracy”, while the Gemenis et al. proposal can be related to ESS questions on political efficacy, attitudes towards democracy, technocratic governance, populism, and authoritarianism. Both proposals covered debates that relate to the COVID-19 pandemic but which would also have relevance beyond it. The proposed items were also felt not to be overly burdensome for respondents to answer and fitted with the wider ESS questionnaire in terms of content and format.

The two sub-modules are summarised below.

### Government authority and legitimacy in the age of a pandemic

The aim of this sub-module is to provide information about how European publics react to pandemic policies. During the pandemic, governments used measures such as stay-at-home orders, business closures, curfews, digital monitoring, and restrictions on movement and assembly. Many of these measures were controversial and some people actively resisted what they believed were unnecessary examples of government overreach. The success of future measures to prevent or contain a pandemic will depend on public support. Therefore, a first very general item asks respondents to what extent they trust their national government to deal with the impact of the coronavirus pandemic (K15).

Many of the most aggressive government policies inflicted considerable economic pain in the interest of protecting public health and thereby increased social and economic inequalities. Relatedly, governments claimed they need extensive (and often unprecedented) power to monitor, surveil, and track the public, in order to enforce compliance with public health measures and to conduct contact tracing for people who test positive for COVID-19. However, liberal democratic societies across Europe also value individual liberty, which may have been threatened by these measures. To study attitudes towards these controversies, several items ask to what extent people prioritise health vs. the economy and government power vs. privacy (K4a, K5a, K4b, K5b). To see to what extent these trade-offs are affected by the pandemic, we included a randomised experimental element where half of the sample was asked to indicate their priorities when fighting a pandemic whereas the other half was asked the same questions without a reference to a pandemic.

Another controversial issue during the COVID-19 pandemic concerned mobility. The pandemic halted travel and migration in an unprecedented way (Piccoli et al., 2021). Asking for views about mobility in light of the pandemic will bring new insight to questions that have motivated scholars for years (De Haas et al., 2020). The sub-module covers both international (K7) and domestic mobility (K8) because fears of COVID-19 spreading from dense cities to suburbs and the countryside brought into play questions about equity between nations, regions and communities. The proposed items ask how important it is to close borders and to restrict people’s movement between different parts of their country when fighting a pandemic (Koopmans, 2020).

Understanding these different opinion dynamics will be essential for governments and other agencies in their struggle to get broad compliance for public health measures. The sub-module will also contribute to our understanding of the future of European democracies, especially when linked to other ESS questions, such as the module on *Understandings and Evaluations of Democracy*. This will allow researchers to connect attitudes towards pandemic measures to broader political and societal issues such as the questions of what powers democratic governments should have and to what extent citizens are willing to tradeoff democratic freedoms for other perceived benefits like health or stability. Further links can be drawn to other topics in the ESS such as the politics of economic and social inequality in Europe, who is viewed as more deserving of government support, and what the priorities should be for contemporary societies. This sub-module intersects with many of those debates by asking about the priorities when fighting a pandemic and the extent to which different societal groups should be privileged (or not).

### COVID-19 conspiracy beliefs and government rule compliance

This sub-module focused on conspiratorial thinking. Conspiracy beliefs can be a major hindrance causing a lack of compliance with public health measures and, more generally, a government’s ability to enforce rules designed to protect the public. The state of the art in conspiracy attitudes lacks the follow through from causes to consequences, especially with a nuanced view of different conspiracies. Understanding the contextual causes of cross-national differences, and the potential consequences, regarding compliance with COVID-19 rules is something we need to help save lives in such a crisis situation. Potential individual causes are tapped well elsewhere in the European Social Survey with questions on institutional trust, trust in government, political efficacy, attitudes towards democracy, life satisfaction, populist attitudes, and one’s approval of technocratic governance (ESS Round 10 questionnaire). To expand on this, we also included trust in scientists (B12a). These potential causes, along with country-contextual characteristics such as economic and political factors or natural experiments emerging from cross-country variations, or more specifically, the impact of the outbreak on people’s lives, will help understand why and how conspiracy theories become more or less prevalent.

Taking inspiration from the Brotherton et al. (2013) conspiracy battery, we measure three dimensions of conspiratorial thinking: general conspiracies, more domain specific-scientific coverup, and finally a COVID-specific item:

1. “A small secret group of people is responsible for making all major decisions in world politics.” (K1)
2. “Groups of scientists manipulate, fabricate, or suppress evidence in order to deceive the public.” (K2)
3. “Coronavirus is the result of deliberate and concealed efforts of some government or organisation.” (K16)

We experimentally explored different response options in two online pilot studies in Austria and the UK comparing a 5-point Likert type response format to a dichotomous measure giving an explicit choice between a conspiratorial and a conventional explanation for an event (cf. Clifford et al., 2019). Aggregation of the two agree and two disagree responses resulted in a similar distribution to the two options of the dichotomous format, while rate of “don’t know” responses corresponded closely to the middle category of the 5-point scale. The Likert scale was deemed preferable given the much lower rate of non-response and greater, potentially meaningful variance making it also usable as a continuous item.

In relation to conspiratorial thinking, relevant outcomes included in the sub-module are policy preferences on health vs economy, border closures, movement restrictions and people’s allowance for government tracking activities in such a crisis situation, and the aforementioned compliance with government rules. To measure the latter, we considered a diverse set of items including attitudes towards social distancing. Given the item “Is it more important for you personally to follow government rules or to make your own decisions when fighting a pandemic?” (K6) was already proposed by Helbling et al. (2020), we settled on only one additional rule-compliance item “Will you get vaccinated against coronavirus with the vaccine that was approved by the national regulatory authority in [country]?” (K20).

## Piloting approach and finalisation of questions

Despite the short period for question development, it was felt crucial to include a stage of pre-testing to help assess question quality. Face-to-face quantitative piloting was not possible due to the lack of time and pandemic-related restrictions in most countries that prevented in-person interviewing. Cognitive interviewing, while desirable, was also not possible within the timescales available. It was therefore decided to carry out a small-scale online quantitative pilot, using a nonprobability access panel.

There are legitimate concerns about the quality of nonprobability online panels (for a debate see Cornesse et al., 2020; Baker et al., 2010). Furthermore, adopting an online approach for testing would differ from the face-to-face approach expected in most countries for Round 10 and any findings may not fully translate between modes. Nevertheless, even this “imperfect” testing would be preferable to no testing, which was the only realistic alternative. Objectives for the online testing included assessing:

- Relative levels of item non-response between items
- Correlations between items
- Comparison of alternative versions of items (allocated to split-ballot samples)

The online testing was found to be very informative in finalising the module. For instance, as noted above, it allowed for different versions of the conspiracy items to be compared experimentally which informed the formulations of the final items. The testing also helped to prioritise items for the final module. For example, a question was included in the testing on intergenerational attitudes (“When jobs are scarce, younger people have more right to a job than older people”). The testing found a very high level of midpoint response for this item (“Neither agree nor disagree”) which strongly contributed to the decision to remove it from the final module. The testing was also delivered in a very short timescale, with around three weeks from commissioning to final data delivery.

The final COVID-19 module questions, finalised following online testing, are included in Appendix C.

## Conclusions and future learning

Despite the challenges described in this article, the experience of developing a COVID-19 module for ESS was a positive one. It demonstrated that in such extraordinary circumstances, it was possible to develop a set of questions for ESS in a far shorter timeframe than is usually allowed for the development of survey modules.

One particular challenge was the need to regularly consult with a large group of ESS National Coordinators on the question design within the short timeframe available to develop the module. The usual ESS timeframes for developing rotating modules may appear generous, but they allow for extensive input from national teams and other key groups, alongside multiple stages of pre-testing. For a cross-national survey like ESS, it is crucial that questions are understood in a comparable way in different countries, cultures, and languages, and the experience of developing the COVID-19 module reinforced the need to carefully plan for this input, even when time is limited.

The online piloting was found to be extremely useful in finalising development of the module, in particular in identifying which items were more problematic and in comparing alternative versions of items. Partly resulting from this experience, a stage of online testing has been added to the development process for the Round 11 rotating modules. This will be included at the start of the testing process and will help to identify any major issues with proposed items at an early stage, in advance of further (and more expensive) later stages of pre-testing.

One limitation of an online quantitative pre-testing method compared with face-to-face testing is the lack of possibly to gain feedback from interviewers, either based on their own experiences or from reactions to questions by respondents. There was an attempt to capture qualitative feedback from respondents in the online testing through a single open question at the end of the survey (“Do you have any comments or feedback on any of the questions included in the questionnaire. For example, anything that was unclear or difficult to answer? Please include any feedback below”). However, this question yielded only very general feedback which was not useful in identifying possible issues with questions. A future learning is that any such feedback questions should be more specific, ideally referring to and following particular questions of interest, in order to yield more useful results (see, for example Behr et al., 2012).

Overall, we were pleased with the decision to adopt this module. It would have been a clear gap for ESS not to collect some additional data on the impacts of the pandemic. The results from the module will not be published until the ESS Round 10 data release, planned for 2022. This may be seen as a weakness at a time where survey data on the pandemic is being delivered on a rapid and frequent basis. However, the focus of the module on longer-term impacts of the pandemic and in the context of a high-quality cross-national study with numerous covariate analysis possibilities should provide valuable additional data for the ESS data users. There have also been requests from other studies to include the items that have been developed, which should extend the benefit of this work beyond ESS.

## Supplementary Information

**Additional file 1.** Appendixes A, B, and C

## Data Availability

The data generated from the ESS COVID-19 module will be available in the ESS Round 10 data. This is expected to be published over two releases in summer and autumn 2022 and available for registered users to download at https://www.europeansocialsurvey.org/data/.

## Abbreviations

AAPOR: American Association for Public Opinion Research
ESS: European Social Survey
ESS ERIC: European Social Survey European Research Infrastructure Consortium
SEAN: Societal Experts Action Network

## Footnotes

1 Cross-national examples of such studies included the COVID-19 Behaviour Tracker by Imperial College London and YouGov, the Ipsos Global Poll, and the Kantar COVID-19 Barometer.

2 See: https://www.europeansocialsurvey.org/methodology/ess_methodology/source_questionnaire/source_questionnaire_development.html for an overview of the ESS questionnaire development process for rotating modules.

3 The question numbers relating to each topic are included in parenthesis. Appendix 3 includes the full module questionnaire.

4 Dropped following piloting.

5 Other surveys reviewed included the COVID-19 Behaviour Tracker by Imperial College London and YouGov, the YouGov COVID-19 Monitor, the Ipsos Global Poll, the Kantar G7 COVID-19 survey, and the Kantar COVID-19 Barometer. The Societal Experts Action Network (SEAN) COVID-19 Survey Archive was also used as a reference source for questions. ESS national teams also provided suggested questions from a range of national surveys.
