# Supplementary material for: Developing a COVID-19 module for the European Social Survey
Source: Meas Instrum Soc Sci. 2021 Nov 23;3(1):9. doi: 10.1186/s42409-021-00029-4 (PMC8609509; doi:10.1186/s42409-021-00029-4)
Supplement: Supplementary file 1 — Additional file 1. Appendixes A, B, and C [file 42409_2021_29_MOESM1_ESM.zip › Appendix A - module development timetable_ESM.pdf]

Appendix A – module development timetable

Date (all 2020)	Task
4 May	Decision taken to include a COVID-19 module for ESS Round 10
5-14 May	Period for ESS national teams to submit proposals for topics to cover in module
21 May	Meeting between ESS Core Scientific Team and national teams to discuss priority topics (ongoing development following this)
22 May	External call for sub-module proposals (5 items) issued
16 June	Deadline for submission of sub-module proposals from external call
29 June	Meeting to select two external sub-modules (5 items each)
30 June	Announcement of selected sub-modules from external call
1 July	Tender issued for agency to provide online access panel testing for module
9 July	Agency appointed for online access panel testing
10 July	Design meeting with the two external sub-module teams to agree questions; further development following this meeting
17 July	All questions confirmed for online testing and sent to agency; translation into Austrian German
27 July – 2 August	Online data collection
3 August	Data from online testing provided
3 – 7 August	Data checking and analysis
10 August	Revisions to questions following online testing agreed; revised questions sent to ESS national teams to review
14 August	Final questions agreed
17 August	COVID-19 module sent to national teams for translation/programming
1 September	Earliest start date for ESS Round 10 fieldwork
