# Supplementary material for: Developing a COVID-19 module for the European Social Survey
Source: Meas Instrum Soc Sci. 2021 Nov 23;3(1):9. doi: 10.1186/s42409-021-00029-4 (PMC8609509; doi:10.1186/s42409-021-00029-4)
Supplement: Supplementary file 1 — Additional file 1. Appendixes A, B, and C [file 42409_2021_29_MOESM1_ESM.zip › Appendix B - full list of topics proposed by national coordinating teams_ESM.pdf]

Appendix B – full list of topics proposed by national coordinating teams

- Whether had COVID-19
- Impact of pandemic on physical health (more broadly)
- Impact on mental health
- Impact of pandemic on employment
- Impact of pandemic on personal/family situation
- Concern, worry and fear about virus
- Extent to which pandemic affects/affected everyday behaviour
- Longer-term impact on behaviour – e.g. travel, shopping, social activities
- Balance between focus on economy and health
- Impact of pandemic on country – economy, democracy, public health, civil liberties, media quality
- Experience of distance learning/home teaching
- Perceptions of measures/restrictions put in place during pandemic
- Knowledge and perceptions about virus (e.g. whether exaggerated or underestimated)
- Trust in scientists
- Trust in different information sources
- Satisfaction with overall government response to pandemic
- Satisfaction with government support for groups particularly affected
- Satisfaction with health services during pandemic
- Trust in government to deal with impact of pandemic
- Democracy and citizens' rights
- Willingness to be vaccinated
- Perceptions of future role of science – e.g. reliance on scientific experts
- Acceptability/willingness to download tracking apps
- How jobs should be prioritised following the pandemic
- International cooperation, movement and relations
