# Supplementary material for: Developing a COVID-19 module for the European Social Survey
Source: Meas Instrum Soc Sci. 2021 Nov 23;3(1):9. doi: 10.1186/s42409-021-00029-4 (PMC8609509; doi:10.1186/s42409-021-00029-4)
Supplement: Supplementary file 1 — Additional file 1. Appendixes A, B, and C [file 42409_2021_29_MOESM1_ESM.zip › Appendix C - final COVID-19 module_ESM.pdf]

Appendix C – final COVID-19 module

ASK ALL
CARD 9 Using this card, please tell me how much you personally trust each of these institutions? Please answer on a score of 0-10 where 0 means you do not trust them at all and 10 means you have complete trust.

B12a. Scientists

**No trust at all**										**Complete trust**	**(Refusal)**	**(Don't know)**
00	01	02	03	04	05	06	07	08	09	10	77	88

SECTION K[1]

ASK ALL

Now some final questions on different topics.

CARD 89 How much do you agree or disagree with the following statements?

K1. A small secret group of people is responsible for making all major decisions in world politics.
K2. Groups of scientists manipulate, fabricate, or suppress evidence in order to deceive the public.

Agree strongly	1
Agree	2
Neither agree nor disagree	3
Disagree	4
Disagree strongly	5
(Refusal)	7
(Don't know)	8

CAPI RANDOMISATION: automated random allocation to Group 1 or Group 2, each comprising approximately half of the sample. K3 should be programmed automatically in the CAPI script and NOT displayed/asked in the interview.

K3 RECORD OUTCOME OF CAPI RANDOMISATION

GROUP 1	1	**GO TO INTRODUCTION BEFORE K4a**
GROUP 2	2	**GO TO INTRODUCTION BEFORE K4b**

[1] This module is optional for countries. It can only be included in countries that are not including any country-specific questions. For countries not including this module, the showcards included here (numbers 89-100) should be removed from the showcard pack.

ASK IF GROUP 1 AT K3

The next set of questions ask about your opinion on different government priorities when fighting a pandemic.

K4a **CARD 90** Is it more important to prioritise public health or economic activity when fighting a pandemic? Please answer on a score from 0-10 using this card, where 0 means it is much more important to prioritise public health and 10 means it is much more important to prioritise economic activity.

**Much more important to prioritise public health**										**Much more important to prioritise economic activity**	**(Refusal)**	**(Don't know)**
00	01	02	03	04	05	06	07	08	09	10	77	88

K5a **CARD 91** Is it more important for governments to monitor and track the public or to maintain public privacy when fighting a pandemic? Please answer on a score from 0-10 using this card, where 0 means it is much more important to monitor and track the public and 10 means it is much more important to maintain public privacy.

**Much more important to monitor and track the public**										**Much more important to maintain public privacy**	**(Refusal)**	**(Don't know)**
00	01	02	03	04	05	06	07	08	09	10	77	88

ASK IF GROUP 2 AT K3

The next set of questions ask about your opinion on different government priorities.

K4b **CARD 90** Is it more important to prioritise public health or economic activity? Please answer on a score from 0-10 using this card, where 0 means it is much more important to prioritise public health and 10 means it is much more important to prioritise economic activity.

**Much more important to prioritise public health**										**Much more important to prioritise economic activity**	**(Refusal)**	**(Don't know)**
00	01	02	03	04	05	06	07	08	09	10	77	88

K5b **CARD 91** Is it more important for governments to monitor and track the public or to maintain public privacy? Please answer on a score from 0-10 using this card, where 0 means it is much more important to monitor and track the public and 10 means it is much more important to maintain public privacy.

**Much more important to monitor and track the public**										**Much more important to maintain public privacy**	**(Refusal)**	**(Don't know)**
00	01	02	03	04	05	06	07	08	09	10	77	88

ASK IF GROUP 1 AT K3

Now some more questions on different government priorities when fighting a pandemic.

ASK IF GROUP 2 AT K3

Now some questions on different government priorities when fighting a pandemic.

ASK ALL

K6 **CARD 92** Is it more important for you personally to follow government rules or to make your own decisions when fighting a pandemic? Please answer on a score from 0-10 using this card, where 0 means it is much more important to follow government rules and 10 means it is much more important to make your own decisions.

**Much more important to follow government rules**										**Much more important to make your own decisions**	**(Refusal)**	**(Don't know)**
00	01	02	03	04	05	06	07	08	09	10	77	88

K7 **CARD 93** Using this card, how important is it for [country] to close its borders when fighting a pandemic?

**Not at all important**										**Extremely important**	**(Refusal)**	**(Don't know)**
00	01	02	03	04	05	06	07	08	09	10	77	88

K8 **STILL CARD 93** Still using this card, how important is it to restrict people's movement between different parts of [country] when fighting a pandemic?

**Not at all important**										**Extremely important**	**(Refusal)**	**(Don't know)**
00	01	02	03	04	05	06	07	08	09	10	77	88

K9 **CARD 94** Overall, how satisfied are you with the [country] government's handling of the coronavirus pandemic? Please answer using this card, where 0 means extremely dissatisfied and 10 means extremely satisfied.

INTERVIEWER: If the respondent cannot answer due to a change of government during the pandemic period, please select "Don't know".

**Extremely dissatisfied**										**Extremely satisfied**	**(Refusal)**	**(Don't know)**
00	01	02	03	04	05	06	07	08	09	10	77	88

CARD 94 Still using this card, please tell me how satisfied you are with the government's response to the coronavirus pandemic in [country] regarding support for the following groups? Please answer on a score of 0-10, where 0 means extremely dissatisfied and 10 means extremely satisfied.

		**Extremely dissatisfied**										**Extremely satisfied**	**(Refu -sal)**	**(Don't know)**
**K10**	People who have experienced job losses or losses of income	00	01	02	03	04	05	06	07	08	09	10	77	88
**K11**	Elderly people in care homes	00	01	02	03	04	05	06	07	08	09	10	77	88
**K12**	Families with school-aged children	00	01	02	03	04	05	06	07	08	09	10	77	88

K13 **STILL CARD 94** Still using this card, please tell me how satisfied you are with the way health services in [country] coped with the coronavirus pandemic and its consequences?

**Extremely dissatisfied**										**Extremely satisfied**	**(Refusal)**	**(Don't know)**
00	01	02	03	04	05	06	07	08	09	10	77	88

K14 **CARD 95** There are different views on how the [country] government balanced protecting the economy and protecting people's health when responding to the coronavirus pandemic. Please say how you think the [country] government responded using the scale on this card.

**They placed far too much importance on protecting the economy and not enough on protecting people's health**					**They got balance about right**					**They placed far too much importance on protecting people's health and not enough on protecting the economy**	**(Refusal)**	**(Don't Know)**
00	01	02	03	04	05	06	07	08	09	10	77	88

K15 **CARD 96** Using this card, please tell me to what extent you trust the national government in [country] to deal with the impact of the coronavirus pandemic. Please answer on a score of 0-10 where 0 means you do not trust the government at all and 10 means you have complete trust.

**No trust at all**										**Complete trust**	**(Refusal)**	**(Don't know)**
00	01	02	03	04	05	06	07	08	09	10	77	88

K16 **CARD 97** Using this card, please tell me how much you agree or disagree with the following statement.

Coronavirus is the result of deliberate and concealed efforts of some government or organisation

Agree strongly	1
Agree	2
Neither agree nor disagree	3
Disagree	4
Disagree strongly	5
(Refusal)	7
(Don't know)	8

K17 **CARD 98** Have you had coronavirus? Please answer using this card.

Yes, I tested positive for coronavirus	1
Yes, I think I had coronavirus but was not tested/did not test positive	2
No, I have not had coronavirus	3
(Refusal)	7
(Don't know)	8

K18 **CARD 99** Has anyone living with you had coronavirus? Please answer using this card.

Yes, someone living with me tested positive for coronavirus	1
Yes, I think someone living with me had coronavirus but they were not tested/did not test positive	2
No, no one living with me had coronavirus	3
I have not lived with anyone since the start of the pandemic	4
(Refusal)	7
(Don't know)	8

ASK IF RESPONDENT'S D.O.B IS LATER THAN 1950 AT F3.

K19 **CARD 100** Using this card, please tell me if any of the following happened to you as a result of the coronavirus pandemic? Please include anything that has happened at any time since the start of the pandemic, even if it is no longer impacting you. Select all that apply.

I was made redundant/lost my job	1
The income from my job was reduced	2
My working hours were reduced	3
I was furloughed[2]	4
I was forced to take unpaid leave/holiday	5
None of these	6
I have not been in work at any time since the start of the pandemic	7
(Refusal)	77
(Don't know)	88

ASK ALL

K20a Will you get vaccinated against coronavirus with a vaccine that was approved by the national regulatory authority in [country]?

INTERVIEWER: If respondent says they have only received a first dose of the vaccine, select 'Yes, I already have'.

Yes, I will	1
Yes, I already have	2
No	3
(Refusal)	7
(Don't know)	8

[2] Furloughed in the sense of still formally employed but either not working or only working part-time whilst some or all of your wages were paid by the government (possibly meaning a reduced pay rate overall).
